# Letter to the Editor: Talus Position Correlates With Dorsiflexion Range of Motion Following a Lateral Ankle Sprain

**DOI:** 10.1002/hsr2.70762

**Published:** 2025-04-23

**Authors:** Syed Hassan Ali, Shanza Shakir, Javed Iqbal

**Affiliations:** ^1^ Liaquat University of Medical and Health Sciences (LUMHS) Jamshoro Sindh Pakistan; ^2^ ⁠Nursing Department Hamad Medical Corporation Doha Qatar

Dear Editor,

We have read the article published by Toyooka et al., titled *“Talus Position Correlates With Dorsiflexion Range of Motion Following a Lateral Ankle Sprain: A Cross‐Sectional Study”* (Health Science Reports, 2025) [1]. In this study, the authors ideally highlighted the correlation between anterior talus deviation and dorsiflexion range of motion (DFROM) followed by lateral ankle sprain. There are some limitations that we want to emphasize to further magnify this novel topic;

Firstly, the sample size of the study is only 36 patients, principally consisting of young age, which limits the findings to other age groups with varying physical activity, and also does not clarify the grading of sprain. The Wisthoff et al. cross‐sectional study, which has a sample size of over 36, offers important information about this relationship. Acute lateral ankle sprains occurred in 55 of the 108 participants in the Wisthoff et al. study. DFROM was found to have increased over time, suggesting a trend toward recovery. The study discovered that following an acute lateral ankle sprain, mechanical laxity and DFROM change over time, with significant variations in DFROM between sprain grades. In contrast to those with grade I sprains, participants with grade III sprains showed less DFROM, indicating that more severe sprains may result in more dorsiflexion restrictions because of altered talus positioning [2].

Secondly, this study keenly concentrates on the correlation between the anterior talofibular ligament (ATFL) and DFROM through an MRI assessment; it did not give ideas about other ligaments like the posterior talofibular ligament (PTFL) and calcaneofibular ligament (CFL). Ultrasound and dynamic assessments can evaluate the correlation between talus position and dorsiflexion range of motion after a lateral ankle sprain. The effects of ligament injuries on ankle mechanics are revealed by these methods. Ultrasound allows a clear view of the lateral ligament complex and other lesions, which is essential for understanding talus position changes and dorsiflexion range of motion. Ultrasound can detect ATFL, PTFL, and CFL injuries, which affect ankle stability and talus position. Ultrasound can detect isolated ATFL injuries and those with ligament injuries, giving a complete ankle assessment [3].

Furthermore, this study did not determine the intensity of pain. Studies examining the impact of mobilization techniques on ankle sprains have shown that the Visual Analog Scale (VAS) is commonly used to numerically assess the intensity of pain during dorsiflexion movements. This scale is frequently used to gauge how painful dorsiflexion movements are for people with lateral ankle sprains. It offers a measurable indicator of pain, making it possible to evaluate how much pain has changed after mobilization and other interventions [4].

In addition, this study evaluates the talus deviation only in non‐weight bearing or unstressed positions. When a patient has a lateral ankle sprain, weight‐bearing DFROM tests, like the weight‐bearing lunge test, are essential for identifying limitations that might not be noticeable in non‐weight‐bearing circumstances. This is so because functional movement patterns and joint stability after an injury are more accurately reflected under weight‐bearing conditions. Weight‐bearing DFROM in injured limbs can be considerably reduced when compared to uninjured ones, according to studies, even when non‐weight‐bearing DFROM seems normal [5].

Lastly, this study did not mention the long‐term complications of talus anterior deviation like chronic ankle instability (CAI), anterior ankle impingement syndrome, and posttraumatic osteoarthritis. The degenerative changes linked to chronic lateral ankle instability may be exacerbated by altered kinematics in the talus position. One possible explanation for the increased incidence of joint cartilage lesions in chronic ankle instability is increased talar anterior deviation. Previous studies have shown a correlation between early onset of posttraumatic osteoarthritis and lateral ankle instability. Several researchers have hypothesized that changing kinematics and cartilage loading may account for osteoarthritic lesions on the medial talus in chronic ankle instability [6]. Also, there is a lack of effectual interventions like manual therapy which helps to improve dorsiflexion and to restore the normal talus position.

In conclusion, we are thankful to the author for giving us the privilege for further investigation on this appreciable topic to enhance the findings regarding the limitations. Future research is recommended to further amplify this study.

## Author Contributions

**Syed Hassan Ali:** conceptualization, data curation, formal analysis, resources, and writing – original draft. **Shanza Shakir:** formal analysis, data curation, resources, writing – original draft, writing – review and editing. **Javed Iqbal:** writing – original draft, writing – review and editing.

## Ethics Statement

The authors have nothing to report.

## Consent

The authors have nothing to report.

## Conflicts of Interest

The authors declare no conflicts of interest.

## Data Availability

The authors have nothing to report.
